# Mucinous cystadenoma of the appendix with enterocutaneous fistula: a therapeutic dilemma

**DOI:** 10.1093/gastro/gou052

**Published:** 2014-08-06

**Authors:** Nawal K. Jha, Dipendra K. Sinha, Abhinav Anand, Mrigendra K. Rai, Anjana Gandhi, Jitin Yadav, Sanjay K. Yadav

**Affiliations:** General Surgery, Rajendra Institute of Medical Sciences, Ranchi, India

**Keywords:** appendiceal tumours, mucinous cystadenoma, psuedomyxoma peritonei

## Abstract

Mucinous cystadenoma of appendix is a rare clinical entity with very few reported cases in the literature. Consensus on optimal surgical management has not been reached. We report the case of a 65-year-old female patient who presented with fistula over the right iliac fossa. Computed tomography (CT) of the abdomen suggested abscess of the parietal wall. Upon exploration, a mass was found to be arising from the tip of the retroperitoneal appendix and the retroperitoneum was studded with mucoid material. Appendectomy was carried out and final histopathology revealed mucinous cystadenoma with no evidence of malignancy. The patient was discharged uneventfully. The unusual presentation of this disease, as retroperitoneal psuedomyxoma without any intraperitoneal pathology, prompted us to report this case.

## INTRODUCTION

Tumours of the appendix are very rare and the usual mode of diagnosis is incidental means most patients operated for appendicitis and if specimen sent for HPE, few will be having neoplastic changes following histopathological examination of an appendectomy specimen done for appendicitis. According to a report published by the National Cancer Institute USA using the Surveillance, Epidemiology and End Results (SEER) database, appendiceal neoplasms account for approximately 0.4% of gastrointestinal tumours [1]. Even though these tumours are rare, they are histologically diverse—mainly mucinous epithelial neoplasms, also called adenomas, cystadenoma, and benign neoplastic mucocele.

Primary mucinous neoplasms of the appendix are found in less than 2% of surgically resected appendices [2]. Mucoceles that are less than 2 cm in diameter are usually simple retention cysts, while hyperplastic epithelium, cystadenoma and cystadenocarcinoma are more likely to be larger than 2 cm [3]. In developing countries, where non-availability of frozen section is a limitation, optimal management of mucinous adenomas is debatable, some surgeons advocating appendectomy only while others prefer right hemicolectomy. We report a rare case with unusual presentation as enterocutaneous fistula and retroperitoneal psuedomyxoma.

## CASE PRESENTATION

**Figure 1.**
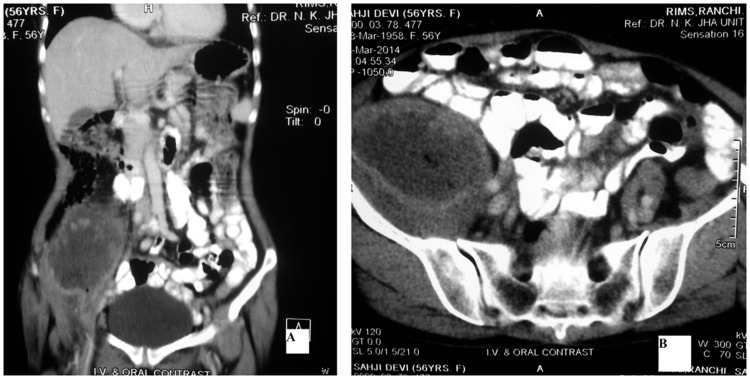
Abdominal computed tomography scans. Coronal view (A) and axial view (B) showing an ill-defined non-enhancing lesion approximately 12.5 × 8.0 × 7.5 cm in size in the right lumbar and iliac region, involving the anterior abdominal wall in its lower part, with air foci within it.

**Figure 2.**
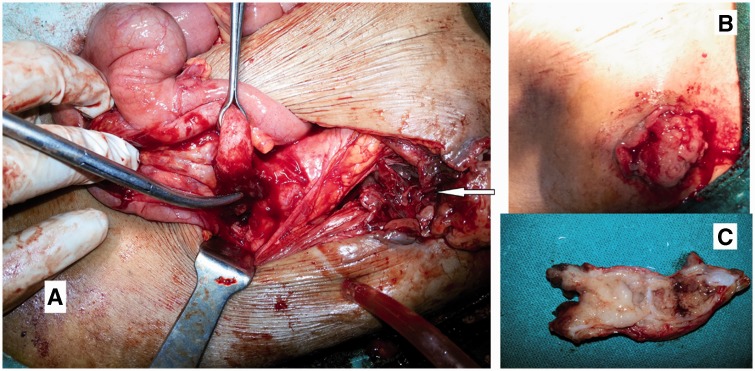
Operative pictures. (A) A retroperitoneal appendix and mass arising from its tip were found. The arrow indicates an iatrogenic fistula created after the incision and drainage procedure. (B) A pre-operative picture of the fistula over the right iliac fossa. (C) Appendectomy specimen with mucoid material.

**Figure 3.**
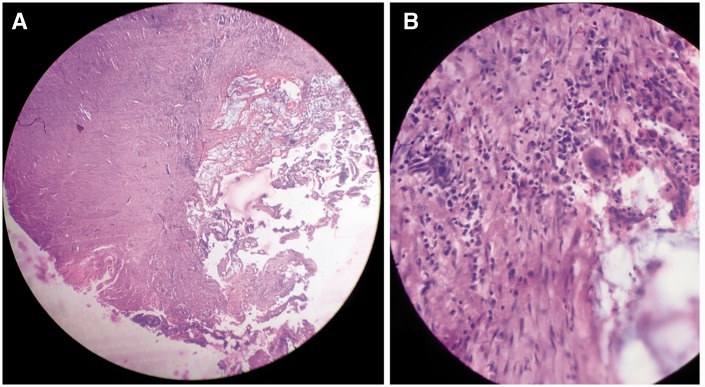
Histopathological pictures (H&E staining). (A) Mucin-secreting Epithelium lining appendix with accumulated mucin (magnification ×4). (B) No deeper tissue invasion or nuclear atypia (magnification ×10).

A 65-year-old female patient was admitted with complaints of a pus-discharging wound over the right inguinal region for a duration of 15 days. A local doctor misdiagnosed the swelling over the right iliac fossa as an abscess and tried an incision and drainage procedure, leading to the formation of a fistula. She was then referred to us. On examination she was stable and afebrile. There was pus mixed mucus discharging wound over the right inguinal region, There was a wound discharging a mixture of pus and mucus with a firm lump 8 × 7 cm in size, palpable in the right iliac fossa. Abdominal computed tomography (CT) showed an ill-defined non-enhancing lesion approximately 12.5 × 8.0 × 7.5 cm in size in the right lumbar and iliac region, involving the anterior abdominal wall in its lower part with air foci within it (Figure 1). The wound was explored under spinal anaesthesia and extended into the retroperitoneum, which was studded with mucoid material; a growth was seen to be arising from the tip of the retroperitoneal appendix (Figure 2). The abdomen was opened through lower midline incision but there was no intra-abdominal pathology. An appendectomy was carried out and the retroperitoneum was washed with normal saline and drainage applied. Histopathological evaluation of a specimen showed tall, columnar, hyperplastic mucosa with focal papillary architecture lining the appendix (Figure 3). Foci of dystrophic calcification, extensive areas of fibrosis and patchy acute and chronic non-specific inflammation were also seen in the wall of the appendix. Mucinous material was observed in the dilated appendicular lumen. There was no evidence of malignancy. An abdominal CT after 3 months showed no sign of recurrence of psuedomyxoma. The patient is in regular follow-up and doing well.

## DISCUSSION

The vermiform appendix is lined with mucous-secreting columnar cells. Changes to the columnar cell layer cause the four following pathologies: (i) retention cyst, (ii) villous hyperplasia, (iii) cystadenoma and (iv) cystadenocarcinoma. All of these changes lead to the formation of a mucocele, which is defined as a dilation of the appendix due to excessive mucinous production. In a classification scheme devised by the American Joint Commission on Cancer (AJCC) and the World Health Organization (WHO) in 2010, lesions limited to the appendix are referred to as adenomas (mucinous), regardless of the cytological appearance or the status of the surgical margin. Only when a tumour is seen within the appendiceal wall is the lesion referred to as invasive mucinous adenocarcinoma [4, 5].

The usual mode of presentation is with features of appendicitis, but mucinous cystadenoma of the appendix can be asymptomatic or may present with an abdominal mass, rectal bleeding, obstruction of the ureter, haematuria or intussusceptions [6, 7]. It has been suggested that abdominal ultrasound does not increase the rate of correct diagnosis [8]. Depending on the contents, well-encapsulated purely cystic lesions with anechoic fluid, hypoechoic masses with fine internal echoes, or complex hyperechoic masses can be seen [9]. Both CT and magnetic resonance imaging (MRI) are useful additional examinations. MRI, in particular, can be used to depict local inflammation and differentiate cysts from the female reproductive organs to exclude the possibility of ovarian cysts. CT of the lower abdomen usually shows a dumbbell-structured, cystic and well-encapsulated mass, including mural calcification in less than 50% [10].

Due to its comparative rarity, the treatment of this tumour is controversial. While it is clear that surgical resection is favoured, the extent of surgery has been called into question. Laparoscopic appendectomy and resection of the appendix with the cecum have been shown to confer a faster post-operative recovery course than the open approach [11]. A simple retention cyst, an appendix with epithelial hyperplasia, and a cystadenoma with an intact base are treated by simple appendectomy. Cystadenomas with a larger base of implantation are an indication for resection of the cecum. Right hemicolectomy used to be the standard treatment for cystadenocarcinomas, but studies have proven that there are no survival advantages with this procedure. A right hemicolectomy is indicated only if one of the following three criteria apply: (i) the need to clear the primary tumour or achieve complete cytoreduction, (ii) lymph node involvement or (iii) histopathological examination indicating a non-mucinous type of cyst [12].

Our patient is in regular follow-up and doing well. Long-term follow-up will help to ascertain the safety profile of the approach we employed, in the absence of the availability of frozen section. In economically disadvantaged countries, the choice of surgery depends on the surgeon’s clinical acumen. Due to the rarity of this disease process, no prospective, randomized trials can definitively elucidate the ideal surgical approach. When a cystic mass in the appendix is seen during operation, appendiceal mucocele should be kept in mind and possible concomitant malignancies should be looked for.

**Conflict of interest:** none declared.
